# A National Behavioral and Clinical Surveillance System of Adults With Diagnosed HIV (The Medical Monitoring Project): Protocol for an Annual Cross-Sectional Interview and Medical Record Abstraction Survey

**DOI:** 10.2196/15453

**Published:** 2019-11-18

**Authors:** Linda Beer, Christopher H Johnson, Jennifer L Fagan, Emma L Frazier, Margaret Nyaku, Jason A Craw, Catherine C Sanders, Ruth E Luna-Gierke, R Luke Shouse

**Affiliations:** 1 Centers for Disease Control and Prevention Atlanta, GA United States

**Keywords:** HIV, public health surveillance, population surveillance, epidemiological monitoring, epidemiology

## Abstract

**Background:**

The Medical Monitoring Project (MMP) is a national population-based behavioral and clinical surveillance system of adults with diagnosed HIV in the United States, and it is sponsored by the Centers for Disease Control and Prevention (CDC). Its purpose is to provide locally and nationally representative estimates of factors affecting HIV transmission risk and clinical outcomes.

**Objective:**

This study aimed to describe the rationale for and methodology of the MMP, in addition to its contribution to evaluating and monitoring HIV prevention, care, and treatment efforts in the United States.

**Methods:**

MMP employs a stratified 2-stage sample design to select annual samples of persons living with diagnosed HIV from the National HIV Surveillance System and conducts interviews and medical record abstractions with participating persons.

**Results:**

MMP data are published routinely via annual reports, conference presentations, and scientific publications. Data may be accessed upon request from the CDC, contingent on the guidelines established for the security and confidentiality of HIV surveillance data.

**Conclusions:**

MMP is the only source of annual population-based data on the behaviors and clinical care of persons with diagnosed HIV in the United States. It provides essential information for monitoring progress toward national treatment and prevention goals and guiding efforts to improve the health of persons with diagnosed HIV and prevent HIV transmission.

**International Registered Report Identifier (IRRID):**

RR1-10.2196/15453

## Introduction

### The Medical Monitoring Project

The Medical Monitoring Project (MMP) is a national population-based behavioral and clinical surveillance system of adults with diagnosed HIV in the United States. It is sponsored by the Centers for Disease Control and Prevention (CDC) and conducted in health departments in 16 states and Puerto Rico, including 6 separately funded cities located within the states. MMP project areas include the following jurisdictions: California; Chicago, Illinois; Delaware; Florida; Georgia; Houston, Texas; Illinois; Indiana; Los Angeles County, California; Michigan; Mississippi; New Jersey; New York City, New York; New York State; North Carolina; Oregon; Pennsylvania; Philadelphia, Pennsylvania; Puerto Rico; San Francisco, California; Texas; Virginia; and Washington.

The primary objectives of MMP are to provide locally and nationally representative estimates of HIV transmission risk behaviors and clinical outcomes among persons with diagnosed HIV; describe health-related behaviors; determine accessibility and use of prevention, care, and support services; increase understanding of care and treatment provided; and examine variations of these factors by respondent characteristics.

### Background

From 2007 to 2014, MMP’s study design relied on a multistage probability sample of persons with diagnosed HIV who were receiving HIV medical care to generate locally and nationally representative estimates of clinical outcomes and HIV-related behaviors [1-4]. With this design, MMP provided data for important national HIV prevention indicators among persons in care for HIV, such as the proportion of those who were prescribed HIV antiretroviral therapy (ART), were adherent to ART, and achieved viral suppression.

Although the importance of ART for reducing morbidity and mortality for persons living with HIV has long been established [5,6], in recent years, the role of ART in HIV prevention has grown more central. When taken as prescribed, ART can reduce the amount of HIV in the blood (viral load) to undetectable levels [7-11]. A person living with HIV who has an undetectable viral load has effectively no risk of transmitting HIV to their HIV-negative sexual partners [12]. Mathematical models show the potential for halting the spread of HIV through an aggressive program of universal testing and immediate ART initiation, a strategy initially dubbed *test and treat* and now more generally known as *treatment as prevention* (TasP) [13-15]. National HIV prevention goals identify 2 areas for critical focus: *broad support for people living with HIV to remain engaged in comprehensive care, including support for treatment adherence* and *universal viral suppression among people living with HIV* [16].

However, from 2007 to 2014, MMP’s design excluded persons with diagnosed HIV who were not receiving HIV medical care, which is necessary to initiate ART and maintain an undetectable viral load. This design limited MMP’s capacity to monitor progress toward linkage to and retention in care objectives and its ability to elucidate barriers to receipt of HIV medical care. In a 2012 report discussing implementation of the US National HIV/AIDS Strategy, the National Academy of Medicine (formerly known as the Institute of Medicine) concluded, “Primary barriers to optimal outcomes for people living with HIV include late diagnosis, delayed linkage to care for HIV, poor retention in care, delayed initiation of ART, and poor adherence to ART…” and recommended MMP expands its population of inference to include HIV-positive persons not receiving medical care [17]. Fortunately, by this time, the National HIV Surveillance System (NHSS)—a CDC-funded surveillance system that monitors national trends in HIV infection diagnoses [18]—had established name-based HIV case reporting in all US jurisdictions and could be used as a source for sampling persons with diagnosed HIV.

Therefore, to address the information gaps described above and enhance the usefulness of the data collected, in 2015, MMP implemented revised methods and began to sample persons directly from the NHSS [18] to represent all persons with diagnosed HIV regardless of receipt of HIV medical care. This increased MMP’s capacity to monitor and guide efforts to prevent HIV infection and improve clinical outcomes through available treatment and other interventions.

## Methods

### Design

Beginning with the 2015 cycle, MMP employed a 2-stage sample design to produce annual representative estimates of the sociodemographic, behavioral, and clinical characteristics of adults with diagnosed HIV in the United States. With this design, participating project areas can produce annual representative estimates of these characteristics among persons in their jurisdictions (ie, states, territory, county, and cities), and the national dataset can be used to produce annual nationally representative estimates.

The first stage of sampling involved a one-time geographically stratified random sampling of US states and territories with probability proportional to size based on the estimated total number of persons living with AIDS as reported to the NHSS at the end of 2002. Although the target population for MMP is all persons diagnosed with HIV in the United States, when the first-stage sample was initially drawn, HIV non-AIDS diagnoses were not reportable in all the states; therefore, the estimated number of persons living with AIDS was used as the best available proxy. Using an indirect measure of size at any given sampling stage does not necessarily affect the validity of the statistical estimates derived from the overall sample if the measure is closely correlated with the desired characteristic, as is the case for AIDS and HIV cases.

On the basis of available funding, 20 primary sampling units were selected during the first stage of sampling in 2004. All 20 state/territory health departments selected for the first-stage sample agreed to participate in MMP. In 5 of the selected states, HIV surveillance activities in 6 large cities are funded separately from the rest of the state, and MMP chose to do the same—resulting in a total of 26 MMP project areas. States with separately funded cities collect data from persons sampled in the state who are living outside of the funded cities; these states receive state-level MMP datasets that combine the state and city data to produce estimates that represent the state as a whole. Owing to budget restrictions, beginning in 2009, 3 areas (Massachusetts, South Carolina, and Maryland) were dropped from the MMP project area sample through a random selection process, resulting in 23 project areas representing 16 states and Puerto Rico. MMP has retained a 100% response rate at this first stage of sampling (ie, all sampled jurisdictions participated) since the inception of this surveillance system.

MMP periodically evaluates the continued validity of the first-stage sample of states and territories. An analysis of counts of reported diagnoses of HIV for 2011 showed that the proportional contribution of states to the burden of HIV had not changed considerably from the distribution of AIDS cases on which the initial sample was based, and this relationship still holds for 2015 HIV diagnosis counts. Thus, the design weights reflecting states’ original sampling probabilities were still reasonably close to what they would have been if sampled using reported HIV diagnoses. On the basis of these findings, we concluded that selecting a new first-stage sample was not warranted, and we retained the original sample, thereby preserving operational efficiencies and the ability of participating states to continue generating local estimates of indicators of HIV care and treatment in their jurisdictions. The 23 participating MMP project areas included approximately 72.80% (732,827/1,006,691) of all persons with diagnosed HIV in the United States during 2016.

**Figure 1:**
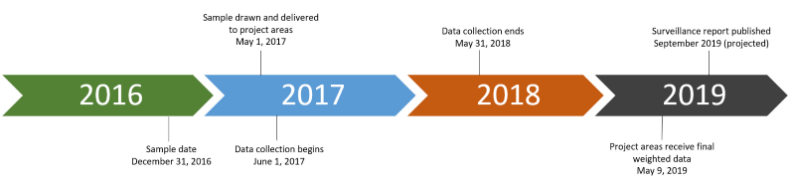
Medical Monitoring Project 2017 cycle timeline.

The second stage of sampling involves annual random sampling of eligible persons directly from the NHSS. A pilot test of these methods was conducted during 2012 to 2014, and findings from that pilot informed the methods used in this study [19,20]. Eligible persons are those who, on the date of sampling, were alive, living with diagnosed HIV, aged ≥18 years, and a resident of an MMP project area. The date of sampling is December 31 of the year before the data collection cycle (eg, December 31, 2014, for the 2015 data collection cycle). Every year, a total of 9700 persons (with minimum state/territory sample size of 400 persons) are selected (Table 1), which allows for estimates of sufficient precision at both the national and local levels. The sample is drawn in March/April of the data collection cycle year to allow time for NHSS reporting delays, and data collection begins in June of the cycle year and ends the following May. For example, the data collection period for the 2015 data collection cycle was from June 2015 to May 2016. Figure 1 presents MMP’s timeline from the date of sampling to the publication of the cycle’s surveillance data report, using outcomes from the 2017 data collection cycle.

**Table 1:** Medical Monitoring Project’s sample sizes by project area.

Project area	Number of persons sampled
California (excluding Los Angeles County and San Francisco)	500
Chicago, Illinois	400
Delaware	400
Florida	800
Georgia	500
Houston, Texas	400
Illinois (excluding Chicago)	200
Indiana	400
Los Angeles County, California	400
Michigan	400
Mississippi	400
New Jersey	500
New York City, New York	800
New York State (excluding New York City)	200
North Carolina	400
Oregon	400
Pennsylvania (excluding Philadelphia)	200
Philadelphia, Pennsylvania	400
Puerto Rico	400
San Francisco, California	400
Texas (excluding Houston)	400
Virginia	400
Washington	400
Total	9700

Health department staff in participating project areas locate and recruit sampled persons using information contained in local health department NHSS records and other available sources such as surveillance databases for other conditions, social services records, and people search engines (eg, Lexis-Nexis, TLO), as permitted by local regulations. Questionnaire data are collected via 45-min telephone or in-person interviews with participating persons, following which a matched medical record abstraction of clinical data is performed for persons who received medical care for their HIV. Questionnaire topics include sociodemographics, HIV treatment and adherence, barriers to care and services, sexual behaviors, alcohol and drug use, stigma and discrimination, met and unmet needs for medical and ancillary services, gynecological and reproductive history, and use of HIV/STD prevention services. Medical record abstraction topics include inpatient and outpatient health care encounters, diagnoses, medications, and laboratory testing and screening. Questionnaires and medical record abstraction instruments are updated approximately every 3 years to improve the quality and accuracy of the data collected and to respond to emerging trends in HIV epidemiology and public health. Participants are given a token of appreciation of approximately US $50 in cash or cash equivalent (eg, gift card), depending on local standards. All sampled persons are offered linkage or reengagement to HIV medical care services, as well as information and referrals for other medical, prevention, and ancillary services, if needed.

As a part of routine public health surveillance, MMP is determined to be nonresearch [21]. Participating states or territories obtain local institutional review board approval to collect data, when required. Informed consent to participate in the project is obtained from all interviewed participants.

The second stage (person-level) response rate for the 2015 cycle, the first year in which new MMP methods were implemented, was 39.8% (3654/9179) after adjustment for eligibility. The response rate for the 2016 MMP cycle was 44.3% (4038/9107), and the response rate for the 2017 MMP cycle was 46.3% (4229/9126). These improvements were because of multiple factors, including the development of more efficient MMP processes and establishment of cooperative relationships needed to successfully find and recruit sampled persons. Although MMP staff work continuously to increase response rates from cycle to cycle, low response rates are not necessarily indicative of nonresponse bias when probabilistic samples are drawn from frames that can provide key information on all sampled persons that can be used to adjust for nonresponse [22], as is the case for the NHSS frame used by MMP. Regardless, as the system matures, we expect improvements in response rates, as was seen in prior years under MMP’s old design.

### Weighting and Data Security

MMP data are first weighted on the basis of known probabilities of selection at the project area and person levels. Then, data are weighted to adjust for nonresponse using known predictors of response based on the NHSS frame. Information available from the NHSS includes age, sex at birth, race/ethnicity, indication of receipt of HIV care as evidenced by laboratory test (CD4 or viral load) results, length of time since HIV diagnosis, completeness of address and phone number information derived from local health department HIV surveillance databases, and mode of HIV acquisition. Using the NHSS as a frame is beneficial because the data are continually updated. An updated frame with the same specifications as the initial frame is drawn 1 year after the construction of the initial frame, which allows MMP data to be adjusted for noncoverage of the population of interest, multiplicity, and updated information on eligibility and HIV care receipt at time of sampling. As a final step in the weighting process, data are poststratified to NHSS population totals for various demographic factors (ie, sex at birth, age, and race/ethnicity) to ensure the data are representative of the population of inference.

MMP data are subject to the CDC’s Data Security and Confidentiality Guidelines for HIV, Viral Hepatitis, Sexually Transmitted Disease, and Tuberculosis programs [23]. These protocols are followed at the project area and national level to ensure the integrity, confidentiality, and security of MMP data. Although local health departments maintain names and contact information for persons with diagnosed HIV reported to the NHSS, no contact information for sampled persons is ever sent to the CDC. MMP itself collects no directly personally identifiable information in its data systems. The software used to collect interview and medical record data has password-protected access so that unauthorized users are unable to view, export, or modify collected data. The security of the system meets all Federal Information Systems Management Act, Office of Management and Budget, Health and Human Services, and CDC Information Technology Security requirements, which ensure the confidentiality, integrity, and availability of data on federal information systems.

MMP collects and monitors data related to core national HIV prevention goals of preventing new HIV infections, increasing access to care, and improving health outcomes for persons living with HIV [16,24]. As MMP includes a range of gender, racial, ethnic, and sexual minority populations, the data collected are also used to assess disparities between groups in these factors. Key MMP estimates used to inform national HIV prevention efforts include ART prescription and adherence, as well as other factors that can affect HIV transmission such as HIV stigma and sexual behaviors. The quality of data collected is maintained by ongoing national and local training of data collectors, the use of electronic data collection systems with built-in logic checks to prevent data entry errors, required data quality assurance activities for MMP project areas, and extensive data quality protocols that govern the processing and cleaning of MMP data.

## Results

### Data Analysis

As mentioned above, in 2015, MMP expanded its population of inference from adults receiving HIV care to all adults with diagnosed HIV regardless of receipt of medical care. This expansion necessitated substantial modification of sampling and weighting methods. As a result of these changes, MMP estimates for 2015 onward are not comparable with those derived using the prior design, and the CDC recommends that analysts do not combine 2015 data with data from prior years or assess trends across the pre- and post-2015 data collection cycles. This recommendation is consistent with the approach of other large national health surveys following methodological changes, such as the Behavioral Risk Factor Surveillance System and the National Survey on Drug Use and Health, which advised against comparing results before and after the implementation of such changes [25,26].

One benefit of MMP’s change to direct sampling of persons from the NHSS is that MMP data are designed to be linkable to NHSS data. This ability to link MMP data to NHSS data allows for additional analyses that can be beneficial for public health programming and service delivery, such as assessing at the individual level whether behaviors that resulted in HIV acquisition continue to be present at the time of interview, which may inform development of tailored HIV prevention interventions among persons living with HIV. In addition, participating state and local health departments can prospectively monitor care access and HIV viral load test results among persons who received linkage or reengagement assistance following the MMP interview.

Owing to MMP’s design, specialized statistical analysis procedures must be used for analysis. When analyzing complex sample data, analysts must consider unequal selection probabilities, nonresponse, and other adjustment factors. Weighted survey procedures in software packages such as SAS and SUDAAN, which require the analyst to specify the design characteristics of MMP, should be used to analyze weighted MMP data. The CDC has prepared documentation for analysts that provides guidelines and sample code for weighted analysis of MMP data.

National MMP data are not publicly available because of the need for specialized technical assistance for working with the large and complex datasets and the security and confidentiality guidelines for the release of HIV surveillance data. However, the CDC will grant access to MMP data in accordance with security and confidentiality guidelines on a case-by-case basis. Researchers may submit analysis concept proposals that are reviewed and prioritized based on their importance for public health, their scientific merit, and on the needs and current workload of the team that oversees MMP at the CDC. There are currently no fees associated with accessing or receiving MMP data, but release is subject to the availability of CDC resources to complete such requests. More information on the appropriate procedures for concept proposals can be obtained by contacting the CDC [27].

For state- or city-level analyses, researchers should coordinate directly with the state or city health departments that conduct MMP in the area(s) of interest. Contact information for the local MMP principal investigators is available on the MMP website [28].

Furthermore, the MMP website provides detailed project information by cycle year, including protocols and data collection instruments [29].

### Data Dissemination

Aggregate national MMP data are published for each data collection cycle in HIV Surveillance Special Reports [30]. These reports provide national estimates of key sociodemographic, clinical, and behavioral characteristics, in addition to information on methods and variable definitions. State and local health departments also regularly publish MMP data via health department reports.

The CDC uses MMP data to guide efforts designed to achieve national goals and objectives set forth in the Division of HIV/AIDS Prevention (DHAP) Strategic Plan and other federal directives [16,24] Specifically, MMP is the data source used by the CDC to monitor homelessness, HIV stigma, and sexual behaviors that increase the risk of HIV transmission among persons with diagnosed HIV. MMP data are also used by the CDC to inform HIV communication campaigns and educational materials (eg, [31,32]).

At the state and city level, MMP data are used to inform the jurisdictions’ Integrated HIV Prevention and Care Plans. These plans are mandatory for certain CDC/DHAP and Health Resources and Services Administration’s HIV/AIDS Bureau grantees and are used to guide HIV prevention and care planning. Specifically, MMP data are used to describe the needs of persons with diagnosed HIV; existing gaps in HIV prevention and care services; and the sociodemographic, behavioral, and clinical characteristics of persons with diagnosed HIV. MMP data inform establishment of priorities, allocation of HIV prevention and care resources, and evaluation of existing programs and policies through its use in local planning processes.

Numerous national and local analyses of MMP data have been disseminated through peer-reviewed scientific journals, through reports, and at national meetings [29]. Publication highlights from recent years include documenting significant improvements in ART prescription and viral suppression among HIV patients [33], increased sexually transmitted disease testing among sexually active HIV patients [34], and an assessment of service delivery and patient outcomes in different medical care settings [35]. In addition, MMP was used as a data source for an influential publication that estimated HIV transmission at each step of the care continuum in the United States, which was published in the *Journal of the American Medication Association Internal Medicine* [36].

## Discussion

MMP is the only source of annual population-based estimates of certain key characteristics among persons with diagnosed HIV needed to assess national and local progress toward US treatment and prevention goals. To advance the CDC’s High Impact Prevention approach to HIV prevention and realize the clinical and prevention benefits of TasP at the population and individual levels, it is critical to ensure that everyone living with HIV is engaged in medical care and virally suppressed. MMP contributes essential information on barriers to treatment and care, use of and adherence to ART, viral suppression, and sexual behaviors that could increase the risk of HIV transmission. National and local MMP data inform geographically tailored approaches to improve HIV treatment and prevention.

## Abbreviations

ART: antiretroviral therapy
CDC: Centers for Disease Control and Prevention
DHAP: Division of HIV/AIDS Prevention
MMP: Medical Monitoring Project
NHSS: National HIV Surveillance System
TasP: treatment as prevention
